# A bioinformatics perspective on molecular classification of diffuse large B-cell lymphoma

**DOI:** 10.1038/s41375-022-01670-6

**Published:** 2022-08-06

**Authors:** Matias Mendeville, Jurriaan Janssen, Yongsoo Kim, Erik van Dijk, Daphne de Jong, Bauke Ylstra

**Affiliations:** Amsterdam UMC Location Vrije Universiteit Amsterdam, Department of Pathology, Cancer Center Amsterdam, Amsterdam, The Netherlands

**Keywords:** Cancer genomics, B-cell lymphoma

## Introduction

The wide range in presentation, treatment response and outcome of diffuse large B-cell lymphoma (DLBCL) reflects a large underlying biological heterogeneity [1]. Various molecular DNA-, RNA- and protein-based subtyping approaches have been proposed over time, but failed to sufficiently capture its biological heterogeneity in a clinically sufficient manner, precluding major clinical consequences [2–5]. The most recent DNA-based subtyping studies as independently proposed by the Dana Farber Cancer Institute (DFCI) and the National Cancer Institute (NCI) are a major step forward [6, 7]. These subtypes are based on DNA-mutation, genome-wide copy number aberration (CNAs), and translocation information. Despite different bioinformatic approaches, the resulting 5- to 7 subtypes largely recognize similar DLCBL pathogenicities and starts to offer a clinically impactful refinement at a level sufficient to serve as a basis for exploration of personalized and targeted treatment in the coming years. Its clinical potential already paid off with the recent finding that benefit from the BTK inhibitor ibrutinib plus R-CHOP is highly specifically associated with two of the genetic subtypes [8]. To enable consistent trial designs and meaningful comparisons between studies, we consider it pivotal to harmonize the currently available DNA-subtyping knowledge into a single classification, preferably widely applicable in diagnostic routine. In this perspective we investigate harmonization opportunities and suggest potential avenues from a bioinformatics point of view.

## Bioinformatics approaches for the current DNA-based DLBCL subtyping

The DFCI and NCI DLBCL subtyping studies are both based on whole exome sequencing data but differ essentially in a priori concepts and bioinformatic strategies. In brief, the DFCI group used unsupervised clustering combined with alteration-centric features. Driver alterations were discriminated from passengers, reducing the genetic dataset to 158 features. Next, unsupervised clustering by means of non-negative matrix factorization (NMF) identified patterns of co-occurring features to define clusters and assign each included patient sample. The NMF algorithm uncovered the optimal stability of subtype clusters to be represented by five groups of similar sizes, which the authors labeled as C1 to C5. The NCI group used semi-supervised clustering combined with gene-centered features. Prior knowledge was used to define four classes with 1 or 2 DNA “seed” features, the a priori assumption. The algorithm subsequently selected additional features with the strongest association to those seeds unsupervised by iteration. All patient samples were included for this 4-class algorithm, but only 46% of cases could be assigned [9]. Recurrent alterations in unassigned cases prompted an extension with two classes. The “seed” features for one of these additional classes were “*TP53* inactivation” and “high CNA load”, in analogy with DFCIs C2 subtype with p53 mutation and deletion (17p) as its top features and a multiplicity of CNAs. This was a first step toward harmonization. The resulting Bayesian-based probability score, named Lymphgen classifier, assigned 63% of cases [7]. Despite the very different designs, most subtypes are remarkably similar with similar underlying biology [1, 7], though some are more similar than others and some are only recognized by one of the algorithms.

## Critical evaluation of the current subtyping systems

Prior to applying their subtyping algorithms, the DFCI and NCI groups used different ways to convert the detected DNA-alterations into features. DFCIs alteration-centric approach regards each DNA alteration type separately be it mutation, translocation, or CNA. Hence, a point-mutation of *CDKN2A*, a deletion at the *CDKN2A*-locus 9p.21 or the entire chromosome 9 arm would each be regarded as separate features. NCIs gene-centric approach combines any DNA-alterations that impact the same gene into a single feature. Hence, any alteration detected that affects *CDKN2A*, would be reduced into a single feature. These two different ways of handling biological features leads to discrepancy in their contribution to subtype assignment that determine biological deregulation and clinical impact. For harmonization we argue that focal chromosomal CNAs which encompass only one or few genes [10] can be readily combined with point-mutations in a gene-centric fashion as these can be assumed to lead to the same overall biological effect [11]. The choice is less obvious for large-scale chromosomal CNAs since these harbor hundreds of genes such that biological insights remain elusive [12] and may be resolved mathematically by calculating an optimal biological characterization of the classes with either feature choice.

Supervised- and unsupervised (machine learning) algorithms may be chosen for subtyping [13]. A supervised approach uses predefined classes to construct a classification rule from the features, while in an unsupervised approach, the algorithm identifies patterns and distinct feature characteristics in unlabeled data. A supervised approach precludes recognition of unknown subtypes. Unsupervised clustering is an elegant data-driven approach that can identify unknown subtypes in high-dimensional data [14–16]. Yet, due to the high number of features unsupervised clustering requires sufficiently large sample sets to recognize rarer subtypes. Rare subtypes are pivotal to recognize since targeted treatment may be available exemplified by 3–4% of *ALK* translocated lung cancers or *ERBB2* positive colon cancers that can be targeted with respectively trastuzumab/neratinib or crizotinib [17, 18]. Likewise, potential specific sensitivity to Ibrutinib of a small fraction of DLBCL patients (<2%) which carry NOTCH1 mutations justifies inclusion as a seed by NCI Lymphgen [8].

Not specifically captured by either of the algorithms are the high-grade B-cell lymphomas (HGBLs) characterized by prognostic features *MYC-* combined with *BCL2* and/or *BCL6* rearrangement [19]. As a solution, NCI Lymphgen introduced a previously published RNA expression-based signature (DHITSig) as a surrogate for *MYC* status as an add-on to the EZB subtype [20]. From a diagnostics point of view this would be suboptimal as it requires two separate assays. Also about 35% of all DLBCLs are assigned as DHITSig-pos whereas genuine *MYC* double- or triple-hits only occur in about 5% of all DLBCL patients [20], indicating that DHITSig is not specific. To resolve the actual relation between DNA-subtyping and HGBL, we argue that unsupervised clustering is the method of choice, whereby the NMF algorithm is attractive given its robustness against the high number of features. However, to enable NMF to recognize a HGBL cluster the number of patient samples should be enlarged with sufficient MYC positive cases and *BCL2* and *BCL6* as features.

**Fig. 1:**
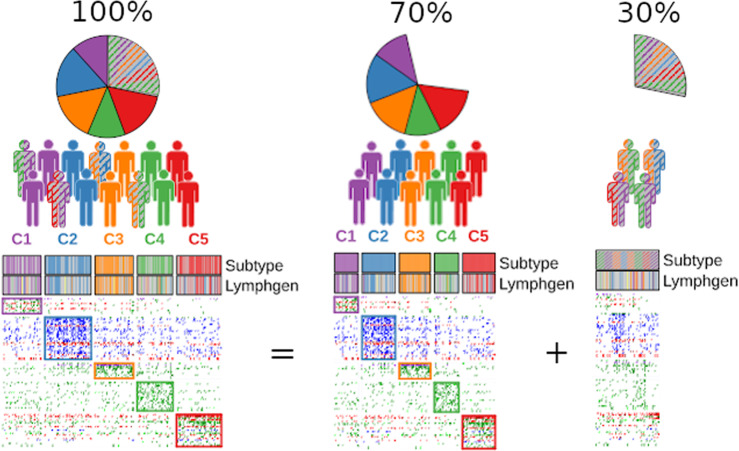
Cluster adherence of DLBCL samples. Cluster adherence was determined using NMF clustering with the 304 DLBCL samples from the DFCI cohort, recapitulating the results from the original study, including five subtypes (C1, C2, C3, C4 and C5) and identical subtype assignment for all samples (left panel). Consensus clustering by resampling [21] illustrates the unstable character of NMF clustering, core patients (solid color, middle panel) and non-core patients (dashed color, right panel). To make this distinction, a stability score was determined by examining co-occurring sample pairs in the same subtype through 1000 iterations of NMF clustering. The heatmaps show patients by column and genomic features by row. Genomic feature colors in the heatmap indicate mutations (green), copy number losses (blue), copy number gains (red) and translocations (purple). DLBCL samples are clustered by subtype. The subtype bars on top indicate core DLBCL samples (colored bars) and non-core DLBCL samples (gray and dashed bars). Lymphgen annotation of the DFCI samples were taken from Wright et al. [7]. Left panel: heatmap of all 304 DLBCL samples from the DFCI cohort. Middle panel: heatmap of the 70% core samples with a high stability score and robust molecular subtypes. Right panel: heatmap of the 30% non-core samples with inconsistent subtype assignment throughout the clustering iterations.

Unsupervised NMF clustering assigns each sample to a cluster, whereas the Lymphgen algorithm assigns samples based on probability, and recognizes that not every DLBCL sample contains sufficient subtype characteristics. A simple exercise of 1000 NMF clustering iterations with each time 80% resampling to determine consensus clustering [21] shows that only about 70% of the DFCI patients are consistently assigned to the same cluster (Fig. 1). The other 30% do not have (sufficient) specific characteristics to be consistently assigned to one or any subtype, like with the Lymphgen algorithm. We believe that this reflects the heterogeneous and continuous nature of DLBCL, supported by recent studies that included mechanistically different mutation-types and thereby further dissect molecular DLBCL classes [22].

While unsupervised clustering is suitable for class identification, ultimately a classifier trained by a supervised algorithm, like the one used in LymphGen, will be required for diagnosis of individual patients, which dictates another step towards harmonization. For training and validation of such parsimonious classification algorithm it will be pivotal to only include consistently assigned samples to eventually provide a classification that is applicable for any DLBCL sample.

## Concluding remarks

Classification for a biologically heterogeneous disease like DLBCL is required for clinical trial inclusion to come to bespoke treatment. To achieve any meaningful classification, there may be well-defined quantitative criteria by which classification schemas can be objectively assessed, but these are inevitably balanced by more subjective choices. We describe here that consensus classification depends on choices concerning the incentive to recognize rare DLBCL subtypes or recognition that not all DLBCLs may have sufficiently specific DNA characteristics to be classified at all. Also, technical choices are to be made such as on the nature and weight of DNA-features, and on mathematics with their pros and cons. Most important is the choice if a consensus classification and a common classifier algorithm is timely and needed. Thereby, we feel that the added value of the achievements of the DFCI and NCI classifications should be exploited by a consensus approach. Arguably, this would be preferred over first evaluating their clinical impact in clinical trials separately or just starting from scratch on yet another classification.

Other translational research groups in the solid tumor arena have met with similar challenges. Probably breast cancer is one of the most successful early examples of an RNA-based classification that found its way into the WHO Classification [23]. Here, international groups converged their biological and bioinformatical approaches through collaboration. Once consensus cell-of-origin classification was achieved and reproducible assays were developed, personalized and targeted treatment could be explored systematically, amongst others in the multiple-armed I-SPY clinical trials [24]. Similarly, a consortium was formed to integrate six independently published RNA-based classifications for colorectal cancer by means of a predefined mathematical approach. The resulting four consensus molecular subtypes are now the basis for various international clinical trials [25].

In our opinion, decisive evaluations of new treatment modalities based on genetics in the heterogeneous disease DLBCL is now largely impeded by a discordancy between the main molecular subtyping approaches. Progress towards personalized treatment of DLBCL would require an international consensus approach for which we have suggested various avenues.
